# Author Correction: Re-designing Interleukin-12 to enhance its safety and potential as an anti-tumor immunotherapeutic agent

**DOI:** 10.1038/s41467-017-02205-9

**Published:** 2018-01-10

**Authors:** Pengju Wang, Xiaozhu Li, Jiwei Wang, Dongling Gao, Yuenan Li, Haoze Li, Yongchao Chu, Zhongxian Zhang, Hongtao Liu, Guozhong Jiang, Zhenguo Cheng, Shengdian Wang, Jianzeng Dong, Baisui Feng, Louisa S Chard, Nicholas R Lemoine, Yaohe Wang

**Affiliations:** 1 Sino-British Research Centre for Molecular Oncology, National Centre for International Research in Cell and Gene Therapy, School of Basic Medical Sciences, Academy of Medical Sciences, Zhengzhou University, Zhengzhou, 450052 China; 2 CAS Key Laboratory of Infection and Immunity, Institute of Biophysics, Chinese Academy of Sciences, Beijing, 100101 China; 3 Department of Cardiology, The First Affiliated Hospital of Zhengzhou University, Zhengzhou, 450052 China; 4 Section of Digestive Diseases, The Second Affiliated Hospital of Zhengzhou University, Zhengzhou, 450014 China; 5 Centre for Molecular Oncology, Barts Cancer Institute, Queen Mary University of London, London, EC1M 6BQ UK

*Nature Communications* 10.1038/s41467-017-01385-8, Article published online 9 November 2017

The originally published version of this Article contained errors in Figure 4. In panel b, the square and diamond labels associated with the uppermost survival curve were incorrectly displayed as ‘n’ and ‘u’, respectively. These errors have now been corrected in both the PDF and HTML versions of the Article.

