# Supplementary material for: Re-designing Interleukin-12 to enhance its safety and potential as an anti-tumor immunotherapeutic agent
Source: Nat Commun. 2017 Nov 9;8:1395. doi: 10.1038/s41467-017-01385-8 (PMC5680234; doi:10.1038/s41467-017-01385-8)
Supplement: Supplementary file 1 — Supplementary Information [file 41467_2017_1385_MOESM1_ESM.pdf]

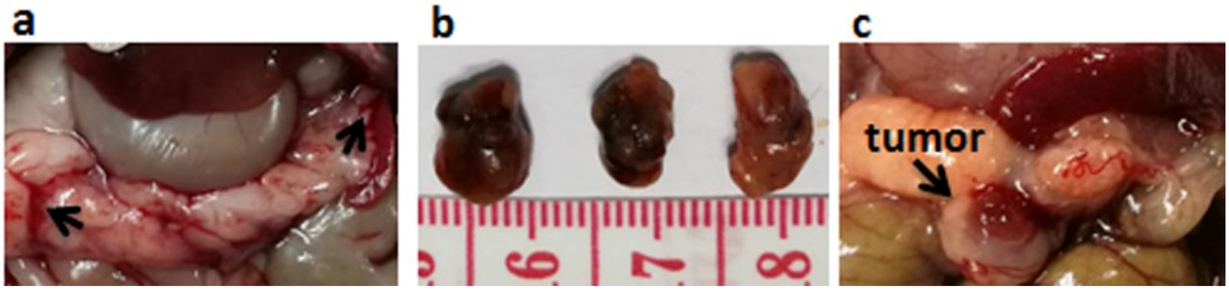

Supplementary Figure 1. Pathological characteristics of pancreatic cancer orthotopic and peritoneally disseminated models in Syrian hamsters.

(**a**) 1x10^{7} SHPC6 cells were seeded into the peritoneal cavity of Syrian hamsters. Four days later, hamsters were anatomized. Left arrow shows the accumulation of serosanguinous ascites fluid. Right arrow shows the multiple oval shaped nodules that had localized and grown in the mesentery adjacent to the pancreas and spleen. (**b** and **c**) Orthotopic PaCa tumors were established in Syrian hamsters using 3×10^{6} Hap-T1 cells. Six days later the pancreas and surrounding tissue was assessed for pathology. There is evidence of substantial tumor growth (**b**) and dissemination from the tumor capsule (**c**).

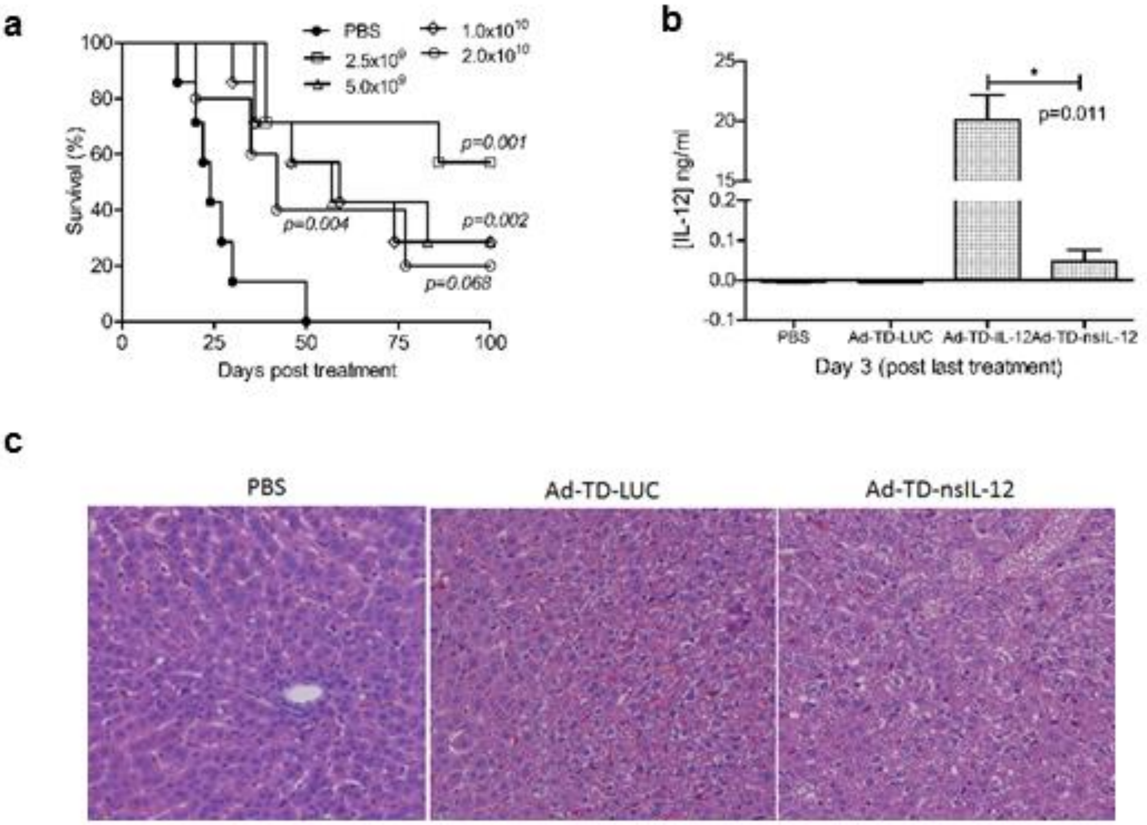

Supplementary Figure 2. Dose escalation of Ad-TD-nsIL-12 suggests that treatment doses of 2.5x10^{9} PFU are sufficient for efficacy. (**a**) 2.5 x10^{9} PFU, 5 x10^{9} PFU, 1 x10^{10} PFU or 2 x10^{10} PFU (n=7/group) Ad-TD-IL-12 were injected i.p into HapT1-bearing animals on days 0, 2, 4, 6, 8 and 10. The survival of hamsters were monitored and Kaplan–Meier survival curves generated. Significance was assessed using the log-rank (Mantel–Cox) test. (**b**) Animals were each injected i.p with 500µl PBS, 2 x10^{10} PFU Ad-TD-LUC, 1 x10^{9} PFU Ad-TD-IL-12 or 2 x10^{10} PFU Ad-TD-nsIL-12 on day 0, 2, 4. Sera were collected on day 3 after last treatment. IL-12 expression in sera was detected by ELISA. Mean plus standard error of the mean is shown. Statistical analysis was carried out using a one-way ANOVA with *post-hoc* Tukey's Multiple Comparison Test or independent-t test. * $p<0.05$. (**c**) Representative histopathology of HE staining of livers after three i.p injections of virus into HapT1-bearing hamsters (n=3/group) on day 0, 2 and 4 at 2 x10^{10} PFU. Livers were collected one day following the last injection and analyzed using H&E staining (original magnification x200).
